# Pedigree Case Report of adult-onset phenotypically heterogeneous Krabbe disease

**DOI:** 10.3389/fgene.2026.1896586

**Published:** 2026-08-24

**Authors:** Lining Chong, Yuanyuan Li, Yali Wang, Jianyu Wang, Jingzhe Han

**Affiliations:** 1 Department of Neurology No.3, Hengshui People’s Hospital, Hengshui, China; 2 Department of Stomatology, Hengshui People’s Hospital, Hengshui, China; 3 Department of Neurology No.1, Hengshui People’s Hospital, Hengshui, China

**Keywords:** adult-onset Krabbe disease, case report, GALC gene, globoid cell leukodystrophy, peripheral neuropathy

## Abstract

**Background:**

Krabbe disease is a rare autosomal recessive leukodystrophy, typically considered a fatal disorder of infancy. Adult-onset forms are uncommon and diagnostically challenging due to their nonspecific presentations.

**Case presentation:**

We report a consanguineous Han Chinese pedigree comprising two siblings diagnosed with adult-onset Krabbe disease, both carrying the identical homozygous GALC c.1048T>G (p.Phe350Val) mutation. Notably, the age at onset differed by 26 years between the two siblings (49 years vs. 23 years), with markedly distinct clinical trajectories—one presenting with progressive dysarthria and peripheral neuropathy, and the other with spastic paraplegia, ultimately leading to wheelchair dependence.

**Conclusion:**

This report describes a family in which identical homozygous GALC mutations presented with markedly distinct clinical phenotypes and disease trajectories during adulthood, thereby substantially expanding the current understanding of the phenotypic spectrum of Krabbe disease and offering novel insights into the differential diagnosis of adult-onset neurodegenerative disorders.

## 1 Introduction

Krabbe disease has traditionally been regarded as a fatal leukodystrophy predominantly affecting infants. However, late-onset forms (onset >2 years of age), particularly adult-onset cases (onset >18 years), are frequently underdiagnosed or misdiagnosed as multiple sclerosis, hereditary spastic paraplegia, or chronic inflammatory demyelinating polyneuropathy due to their rarity and nonspecific clinical manifestations. The GALC gene exhibits a broad mutation spectrum, and certain mutations (e.g., c.1048T>G) are associated with residual enzyme activity that may lead to very-late-onset phenotypes. Nevertheless, the mechanisms underlying the wide inter-individual variability in age at onset—spanning several decades—within the same family pedigree remain unclear. This report describes a family in which identical homozygous GALC mutations presented with markedly distinct clinical phenotypes and disease trajectories during adulthood, thereby substantially expanding the current understanding of the phenotypic spectrum of Krabbe disease and offering novel insights into the differential. The clinical timeline for both patients is summarized in Table 1.

**TABLE 1:** Timeline of clinical course for patients II-2 and II-3.

Time point	Patient II-2 (proband, sister)	Patient II-3 (brother)
Age at onset	49 years	23 years
Initial symptoms	Progressive dysarthria	Progressive limb weakness, difficulty walking
Age at first evaluation	50 years	35 years
Neurophysiology	Demyelinating peripheral neuropathy	Demyelinating peripheral neuropathy (first detected at age 33)
Brain MRI	Normal	Few small ischemic foci
Genetic diagnosis	Homozygous GALC c.1048T>G (p.Phe350Val)	Homozygous GALC c.1048T>G (p.Phe350Val)
Age at genetic confirmation	50 years	35 years
Age at last follow-up	52 years	37 years
Functional status at last follow-up	Dysarthria (speech understandable), ambulatory, normal daily living	Wheelchair-dependent

## 2 Case report

### 2.1 Pedigree members

**FIGURE 1:**
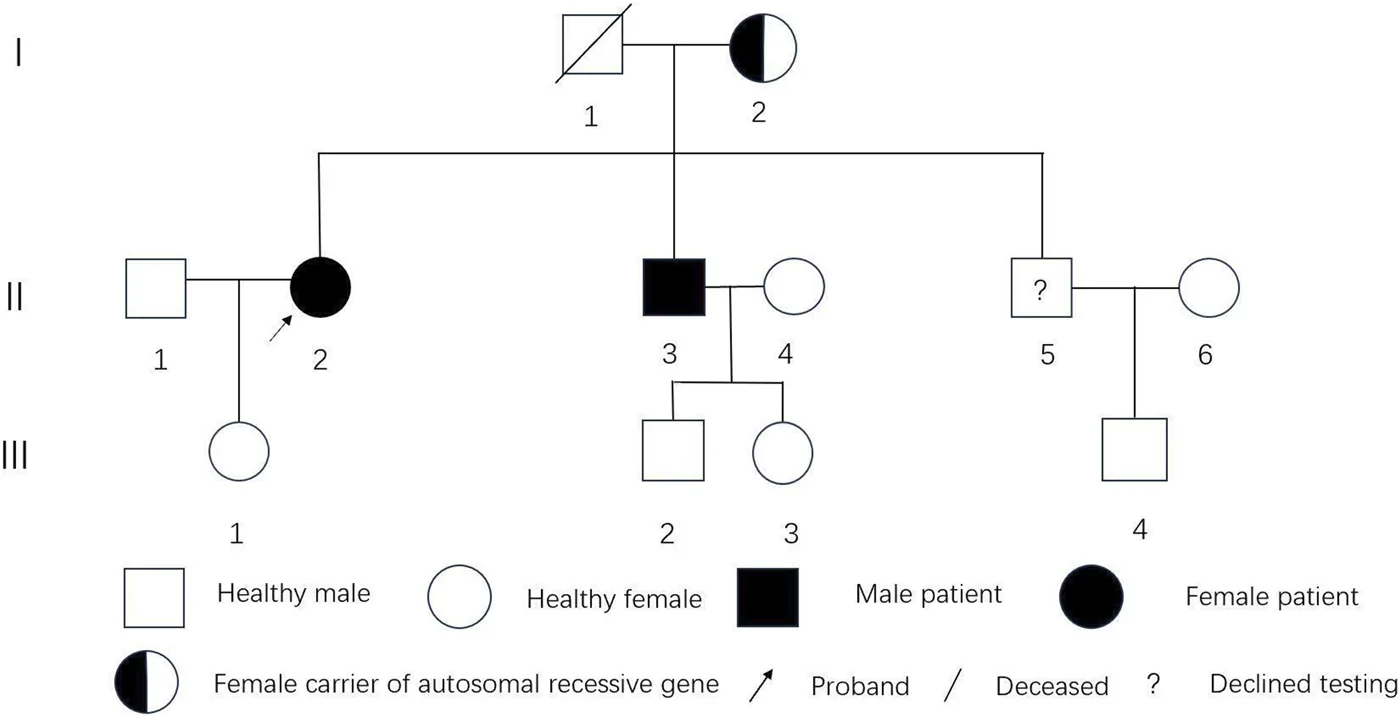
Pedigree of the family with Krabbe disease.

The family members are of Han Chinese ethnicity with a history of consanguineous marriage. The parents (I-1, I-2) are phenotypically normal and had one daughter (II-2, the proband) and two sons (II-3 and II-5) (Figure 1).

### 2.2 Proband (II-2, onset at age 49)

The proband is a female who was admitted to the hospital with a chief complaint of progressive dysarthria for 1 year. The patient began experiencing dysarthria 1 year prior to admission, which has progressively worsened.Past medical history: The patient underwent surgery for papillary thyroid carcinoma 10 years ago and has been regularly taking oral levothyroxine sodium since the operation.Neurological examination: The patient was alert and oriented. Mild dysarthria and tongue muscle atrophy were observed, while the remaining cranial nerve examinations were unremarkable. Atrophy of the fourth dorsal interosseous muscle of the left hand and the thenar muscles of the right hand was noted. The patient also presented with pes cavus deformity.Laboratory investigations: Routine blood, urine, and stool tests, liver and kidney function tests, blood glucose, lipid profile, folic acid, vitamin B12, serum homocysteine, thyroid function tests, autoantibody panel, rheumatologic and immunological screening, antinuclear antibodies (ANA), anti-neutrophil cytoplasmic antibodies (ANCA), tumor markers, tests for HIV, syphilis, hepatitis B and C, serum immunofixation electrophoresis, and serum antibodies associated with peripheral neuropathy were all within normal limits or negative. Galactocerebrosidase activity in leukocytes, measured using the artificial fluorogenic substrate method, was 348 nmol/L/h, corresponding to 33.72% of the normal control value.Cerebrospinal fluid (CSF) analysis: Lumbar puncture revealed an opening pressure of approximately 180 mmH_2_O. Routine CSF analysis, including biochemistry and cytology, showed no abnormalities.Neurophysiological examination: Nerve conduction studies revealed demyelinating peripheral neuropathy.Neuroimaging: Brain MRI revealed no abnormalities. Contrast-enhanced MRI of the brachial and lumbosacral plexuses showed no significant thickening or abnormal enhancement of the bilateral brachial and lumbosacral nerve roots.

### 2.3 Proband’s younger brother (II-3, onset at age 23)

The patient is a 35-year-old male who presented with a 12-year history of progressive limb weakness accompanied by stiffness and difficulty walking. His past medical history was unremarkable.Neurological examination revealed tongue fasciculations and atrophy. Decreased muscle bulk and atrophy were observed in both upper limbs, as well as atrophy of the bilateral quadriceps femoris muscles, more pronounced on the right side. Muscle strength testing revealed: left upper limb proximal 3/5, distal 2-/5; right upper limb proximal 3+/5, distal 2+/5; right lower limb 5-/5; left lower limb 5/5. Muscle tone was decreased in both upper limbs. Hypoesthesia to pinprick and reduced vibratory sensation were noted in the left upper limb below the elbow, predominantly in the ulnar distribution. Biceps and triceps reflexes were absent bilaterally, while bilateral patellar and Achilles tendon reflexes were hyperactive. Bilateral Chaddock and Babinski signs were positive.Neurophysiological examination performed 10 years after disease onset (at age 33) revealed demyelinating peripheral neuropathy.Laboratory investigations: Routine blood, urine, and stool tests, erythrocyte sedimentation rate (ESR), coagulation function tests, antinuclear antibodies (ANA), urinary light chains, immunofixation electrophoresis, folic acid, vitamin B12, vasculitis screening tests, rheumatoid arthritis panel, and immunoglobulins were all within normal limits or negative.Serum antibodies including Hu, Yo, Ri, GM1, VGKC, and anti-Borrelia antibodies were also negative. Galactocerebrosidase activity in leukocytes, measured using the artificial fluorogenic substrate method, was 72 nmol/L/h, corresponding to 6.98% of the normal control value.Cerebrospinal fluid (CSF) analysis: Lumbar puncture revealed an opening pressure of approximately 110 mmH_2_O. Routine CSF analysis and cytology showed no abnormalities. CSF biochemistry revealed a slightly elevated protein level of 0.55 g/L. Tests for RPR, TORCH, Hu, Yo, Ri, GM1, VGKC, and anti-Borrelia antibodies in CSF were all negative.Neuroimaging: Brain MRI revealed a few small ischemic foci. Cervical spine MRI showed no abnormalities. Nerve ultrasound of both upper limbs revealed diffuse swelling and thickening of the bilateral brachial plexuses, median nerves, and ulnar nerves.

Neither patient received disease-specific therapy. At the 2-year follow-up, the proband (II-2, aged 52) remained ambulatory with dysarthria (speech understandable) and continued normal daily living without assistance. Her younger brother (II-3, aged 38) had become wheelchair-dependent due to progressive lower limb weakness.

### 2.4 Genetic analysis

**FIGURE 2:**
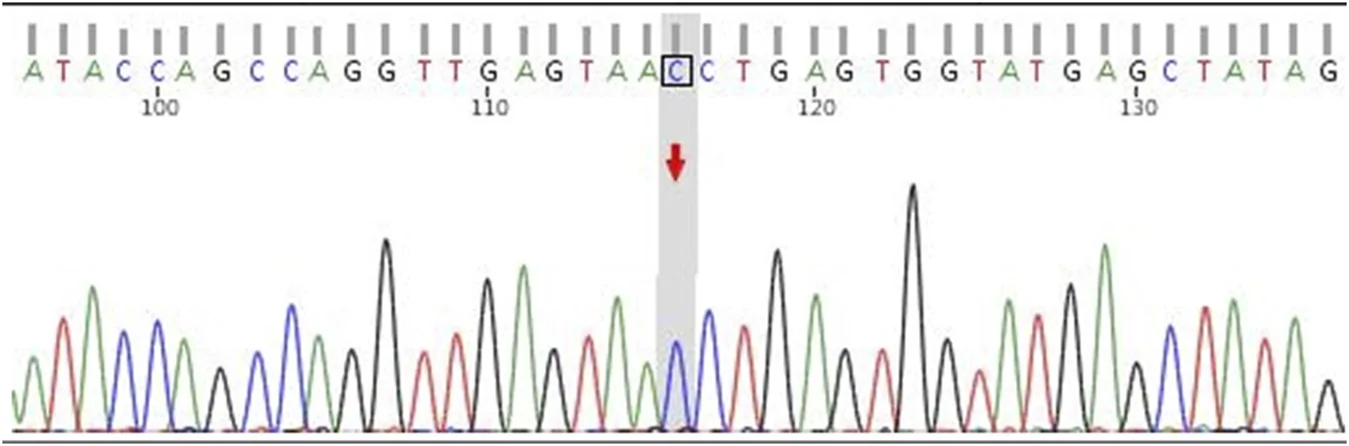
Sanger sequencing chromatogram of the GALC gene in II-2 (the proband). The arrow indicates the homozygous mutation site c.1048T>G (p.Phe350Val).

**FIGURE 3:**
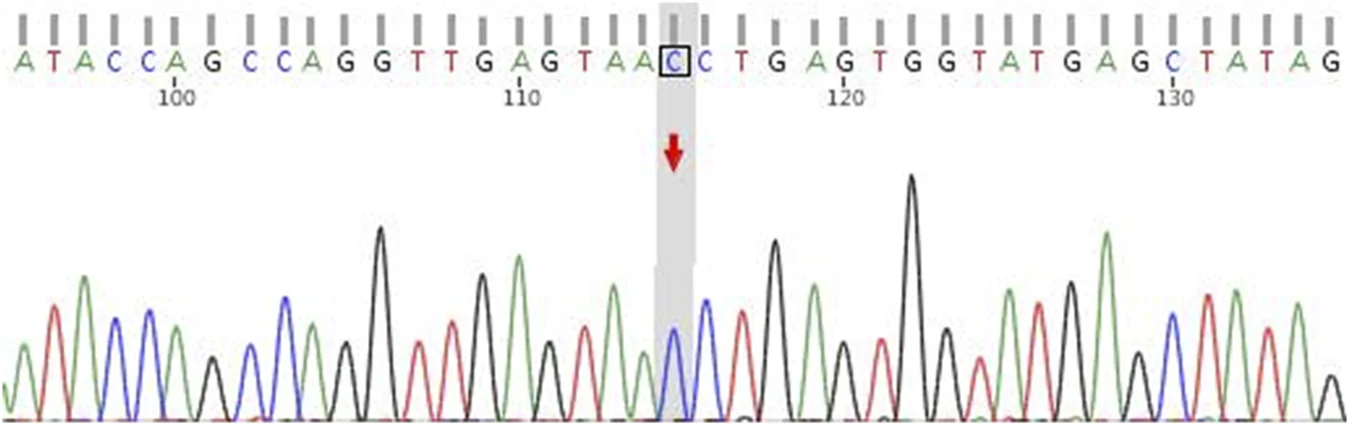
Sanger sequencing chromatogram of the GALC gene in II-3 (the younger brother of the proband). The arrow indicates the homozygous mutation site c.1048T>G (p.Phe350Val).

**FIGURE 4:**
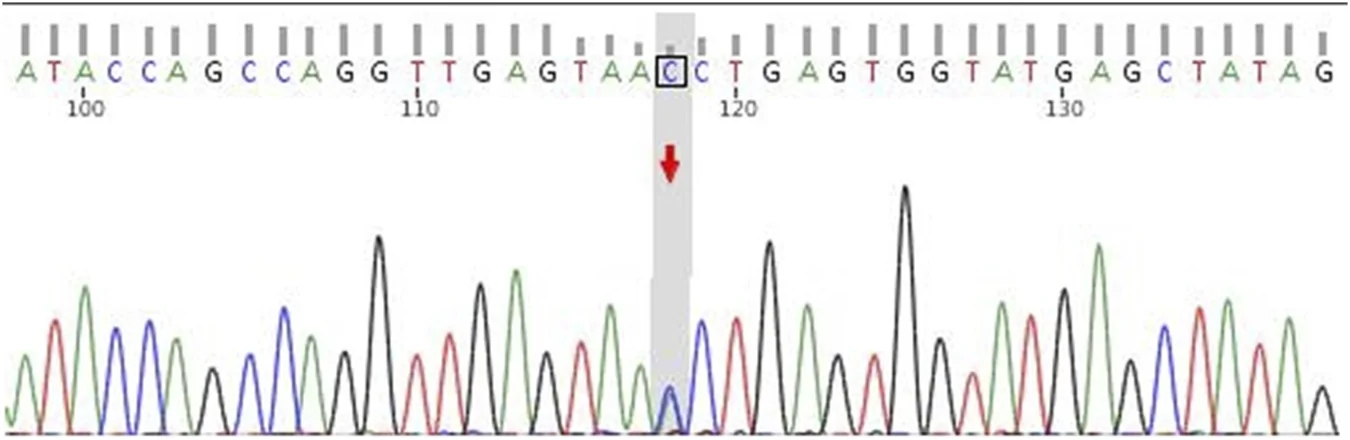
Sanger sequencing chromatogram of the GALC gene in I-2 (the mother of the proband). The arrow indicates the heterozygous mutation site c.1048T>G (p.Phe350Val).

After obtaining informed consent, 5 mL of peripheral blood was collected from II-2, II-3, and their mother for whole-exome sequencing (WES). Individual II-5 declined participation. All examinations were performed with the informed consent of the patients and approval from the Ethics Committee of Hengshui People’s Hospital.High-throughput sequencing and subsequent Sanger sequencing identified a mutation site: the c.1048T>G variant in the GALC gene. Sanger sequencing validation confirmed that both II-2 and II-3 carried a homozygous c.1048T>G (p.Phe350Val) mutation in the GALC gene (Figures 2, 3), while their mother was a heterozygous carrier of the c.1048T>G (p.Phe350Val) mutation (Figure 4). The pedigree is shown in Figure 4.

## 3 Discussion

Krabbe disease is a rare autosomal recessive lysosomal storage disorder first reported by Knud Krabbe in 1916 (Knudkrabbe, 1916). The causative gene is the β-galactocerebrosidase (GALC) gene located on chromosome 14q24.3-q32.1 (Chen et al., 1993). Mutations in this gene result in reduced galactocerebrosidase enzyme activity, leading to damage in the central and peripheral nervous systems (David Wenger et al., 1997).

The clinical manifestations of adult-onset Krabbe disease are highly heterogeneous, and diagnosis remains challenging. Presenting features may include spastic paraplegia, ataxia, peripheral neuropathy, visual impairment, and other symptoms, often leading to misdiagnosis (Bajaj et al., 2002). Cranial magnetic resonance imaging (MRI) plays a crucial diagnostic role, with characteristic findings including T2 hyperintensities in regions such as the precentral and postcentral gyri, corticospinal tracts, optic radiations, and medial lemnisci. Electrodiagnostic studies typically reveal diffuse demyelinating peripheral neuropathy, with axonal involvement being less common (Rabab et al., 2012; Potter et al., 2013). The GALC c.1048T>G (p.Phe350Val) variant was classified according to the ACMG/AMP guidelines (PMID: 27993330), combined with pedigree segregation analysis, and was designated as a likely pathogenic variant based on the following evidence: PM2 (absent from controls in population databases), PM3 (homozygous in affected individuals from a consanguineous family), PP1 (co-segregation with disease in the pedigree), and PP3 (multiple *in silico* tools predict a deleterious effect).

In this study, the affected individuals carried the c.1048T>G (p.Phe350Val) variant, a missense mutation in the coding region of the GALC gene. Analysis of next-generation sequencing (NGS) data from the pedigree revealed that the mother of the proband was a heterozygous carrier of this variant, whereas individuals II-2 and II-3 were homozygous for the variant. This variant has not been reported in the large-scale population frequency database gnomAD. However, it has been documented in multiple patients with Krabbe disease, typically in a compound heterozygous state. Based on the segregation analysis within this pedigree, the variant is classified as pathogenic.

This pedigree presents two extreme and atypical clinical phenotypes of adult-onset Krabbe disease: one case presenting as an insidiously onset, slowly progressive “spastic paraplegia-peripheral neuropathy” syndrome (onset at age 23), and the other characterized by dysarthria and peripheral neuropathy (onset at age 49). Adult-onset Krabbe disease is a “great mimicker.” In this pedigree, the spastic paraplegia-peripheral neuropathy phenotype of individual II-3 could easily be misdiagnosed as amyotrophic lateral sclerosis or hereditary spastic paraplegia (HSP) (Zhang et al., 2021). McCarron et al. reported a 55-year-old male with a novel GALC pathogenic variant who presented with slowly progressive spastic paraparesis, with a diagnostic delay of over 3 years. This parallel underscores that adult-onset Krabbe disease represents a diagnostic challenge with variable expressivity, and that a combination of clinical, imaging, enzymatic, and genetic approaches is essential for timely diagnosis (Mccarron et al., 2025). Whereas individual II-2 requires differentiation from conditions such as chronic inflammatory demyelinating polyneuropathy and other hereditary peripheral neuropathies (Sabatelli et al., 2002). The diagnostic challenges encountered in our pedigree are consistent with the broader issues highlighted by Urizar et al. (2025), who emphasized the difficulties in recognizing highly attenuated lysosomal storage disorders in adult populations. As noted by the authors, the non-specific and slowly progressive nature of these conditions often leads to significant diagnostic delay or misdiagnosis as more common neurological entities. Our cases, presenting with a phenotypic spectrum ranging from spastic paraparesis to peripheral neuropathy, underscore this diagnostic complexity and reinforce the importance of considering lysosomal storage disorders, including Krabbe disease, in the differential diagnosis of adult-onset spastic paraplegia of unknown etiology (Senarathne et al., 2025).

Although the c.1048T>G mutation, resulting in the p.Phe350Val substitution (p. Phe350Val—Note 1), may retain minimal residual enzyme activity or partial function, leading to extremely slow accumulation of the toxic metabolite psychosine that does not exceed the compensatory threshold of the nervous system until mid-adulthood, thereby causing disease onset (Pastores, 2009; Graziano and Cardile, 2015), the fact that the same homozygous mutation resulted in a 26-year difference in age at onset between the two affected siblings suggests that the underlying mechanisms extend beyond a simple “residual enzyme activity” explanation (Verdru et al., 1991). Epigenetic modifications, environmental factors (such as infection or trauma triggers), and genetic modifier genes involved in psychosine metabolism, neuroinflammation, and myelin repair efficiency may collectively determine the “timing” of disease onset (Du et al., 2012; Hassan et al., 2017). The later age at onset in the female sibling suggests that estrogen may also influence GALC activity. Notably, neither patient showed evidence of definite central nervous system involvement on neuroimaging, a relatively uncommon finding in adult-onset Krabbe disease.

This study has several limitations. First, psychosine levels were not measured, which would have provided additional biochemical evidence supporting the diagnosis and could have helped to further delineate the genotype–phenotype correlation. Second, functional assays to assess the impact of the GALC c.1048T>G variant on enzyme function were not performed. However, the diagnosis was unequivocally established by the combination of reduced GALC enzyme activity in leukocytes, the homozygous pathogenic variant confirmed by ACMG criteria, and the characteristic clinical and neurophysiological findings. Future studies incorporating longitudinal biomarker assessments and functional validations are needed to further explore the mechanisms underlying the phenotypic heterogeneity observed in this pedigree.

## 4 Conclusion

We report an exceptionally rare pedigree of adult-onset Krabbe disease caused by a homozygous c.1048T>G mutation in the GALC gene, exhibiting marked intrafamilial phenotypic heterogeneity. This case strongly suggests that adult-onset Krabbe disease may be more common than previously recognized, potentially masquerading as various “idiopathic” adult-onset neurological disorders. Clinicians should maintain a high index of suspicion for Krabbe disease in adult patients presenting with combined central and peripheral nervous system involvement or symmetric pyramidal tract signs, and include it in the routine differential diagnosis with confirmatory genetic testing. Furthermore, once a proband is diagnosed, targeted genetic screening of all first-degree relatives, regardless of age, is essential.

### 4.1 Patient perspective

The 52-year-old proband (II-2) shared her perspective: “When my speech started getting worse, I saw many doctors, but no one could tell me why. Getting a genetic diagnosis finally gave me an answer. I am grateful that I can still live normally and take care of myself. I hope our family story can help other patients get diagnosed earlier and not go through the same uncertainty I did”.

Her 37-year-old brother (II-3) added: “For 12 years, I did not know what was happening to me. My legs became weaker, and walking became harder and harder. Now I understand it is a genetic disease. Even though I need a wheelchair now, knowing the truth brings me some peace. I hope doctors will think of this disease when they see similar cases, so others do not have to wait as long for answers”.

## Data Availability

The datasets presented in this article are not readily available because of ethical and privacy restrictions. Requests to access the datasets should be directed to the corresponding author.
